# White islands in a sea of red

**DOI:** 10.1016/j.idcr.2024.e02072

**Published:** 2024-09-04

**Authors:** Sara Elizabeth Milla Salguero, Eduardo Smelin Perdomo Domínguez

**Affiliations:** a Faculty of Medicine, Catholic University of Honduras, Honduras; b GIMUNICAH, Faculty of Medicine, Catholic University of Honduras, San Pedro Sula, Honduras

**Keywords:** Dengue rash, Dengue fever, Dermatology, Mucocutaneous

## Abstract

Dengue is a viral disease caused by a single-stranded RNA virus from the Flaviviridae family, primarily transmitted by the Aedes aegypti mosquito, although Aedes albopictus also plays a role as a vector. Clinical features of dengue range from nonspecific symptoms to severe forms like dengue shock syndrome. Among these clinical features, dermatological manifestations are particularly noteworthy, as they can aid in differentiating dengue from other illnesses.

## Case illustrated

**Fig. 1:**
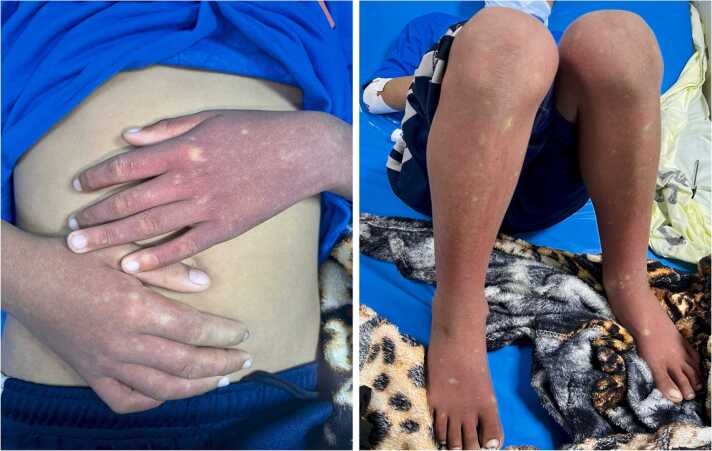
White islands in a sea of red characteristic rash on both the upper and lower extremities.

A 10-year-old male presented to the pediatric emergency department with a five-day history of fever, followed by two days of defervescence, accompanied by vomiting, mild abdominal pain, and a diffuse rash. Vital signs were normal. On examination, a diffuse, confluent, non-blanching erythematous rash was observed on the upper and lower extremities (Fig. 1), with macules and patches of normal skin, resembling "white islands in a sea of red." Initial laboratory tests showed hematocrit 66.6 %, platelets 37,000/mm³ , and leukopenia. Positive nonstructural protein 1 antigen and IgM confirmed dengue diagnosis. Initial management included intravenous fluid support. Follow-up tests reported hematocrit 38 % and platelets 72,000/mm³ .

Dengue is caused by a single-stranded RNA virus from the Flaviviridae family, primarily transmitted by the Aedes aegypti mosquito [1]. The clinical manifestations of dengue range from high fever, myalgias, and arthralgias to severe forms like dengue shock syndrome. Additionally, dengue often presents with a variety of dermatological signs, including cutaneous eruptions, which have been reported in 46.8–82 % of cases according to observational studies [2]. Dengue infection can cause a confluent, non-blanching erythematous rash with macules and patches of normal skin, referred to as "white islands in a sea of red." This type of rash usually appears during defervescence and gradually fades over the course of a week. Various factors are believed to contribute to the development of this rash, including viral replication in immune cells like Langerhans cells in the skin, direct skin infection by the dengue virus, and complex interactions between the virus and the host's immune response [3].

The characteristic "white islands in a sea of red" rash in dengue is a useful diagnostic clue, indicating increased capillary permeability and fluid leakage [4]. This distinctive rash is a hallmark of dengue defervescence and aids in distinguishing dengue from other febrile illnesses with similar presentations. The patient improved and was discharged after completing recovery without complications. The rash persisted at discharge and resolved gradually within three days.

## Ethics statement

Informed consent was obtained from the patient's legal representative and all procedures were performed according to the Declaration of Helsinki.

## Funding sources

This research did not receive any specific grant from funding agencies in the public, commercial, or not-for-profit sectors.

## Consent

Written informed consent was obtained from the patient to publish this report in accordance with the journal's patient consent policy.

## Declaration of Competing Interest

The authors declare that they have no known competing financial interests or personal relationships that could have appeared to influence the work reported in this paper.

## References

[1] Paz-Bailey G., Adams L.E., Deen J., Anderson K.B., Katzelnick L.C. (2024). Dengue. Lancet.

[2] Srivastava A. (2017). Dengue fever rash: white islands in a sea of red. Int J Dermatol.

[3] Thomas E., John M., Kanish B. (2010). Mucocutaneous manifestations of dengue fever. Indian J Dermatol.

[4] Gautam G., Khera D., Singh K. (2020). Isles of white in a sea of red: an underdiagnosed entity?. BMJ Case Rep CP.

